# COVID-19 and Acute Coronary Syndrome: A Literature Review

**DOI:** 10.7759/cureus.29747

**Published:** 2022-09-29

**Authors:** Abidemi O Akinrinmade, Vivien O Obitulata-Ugwu, Nkechi B Obijiofor, Folami Victor, Mimidoo Chive, Farirai M Marwizi, Lilian O Odion-Omonhimin, Nmachi B Obasi

**Affiliations:** 1 Medicine and Surgery, Benjamin S. Carson School of Medicine, Babcock University, Ilishan-Remo, NGA; 2 United States Medical Licensing Examination (USMLE) Education, Kaplan Medical Prep, Manhattan, USA; 3 College of Medicine, University of Nigeria, Enugu, NGA; 4 Medicine and Surgery, Nnamdi Azikiwe University, Awka, NGA; 5 College of Medicine, Grodno State Medical University, Grodno, BLR; 6 Infectious Disease, Richmond Gabriel University, Arnos Vale, VCT; 7 Cardiology, Universitatea de Medicina si Farmacia din Timisoara, Timisoara, ROU; 8 Medicine and Surgery, University of Benin, Benin, NGA; 9 Cardiology, Rivers State University Teaching Hospital, Port Harcourt, NGA

**Keywords:** st-segment elevation myocardial infarction, sars-cov-2, non–st-segment elevation myocardial infarction, covid-19, acute coronary syndrome

## Abstract

The coronavirus disease 2019, also known as the COVID-19 pandemic has had a deleterious impact on daily living, with health and socioeconomic effects of a global magnitude. Acute coronary syndrome (ACS), an important cardiovascular disease with significant morbidity and mortality rates, has been frequently reported in patients with this novel virus. This review aims to discuss the potential associations between COVID-19 and ACS with the use of multiple databases, including but not limited to; PubMed, ScienceDirect, World Health Organization, and American Heart Association.

We have explored the pathophysiology of ACS, focusing on COVID-19 in particular with the use of various works of literature that highlights the pattern of viral entry and replication via the angiotensin-converting enzyme II. The review has also discussed the impact of the pandemic on hospital admissions, diagnosis, and management of ACS patients, as well as briefly highlighted a possible link between the widely available COVID-19 vaccines and possible cardiovascular complications.

The association between COVID-19 and ACS needs more in-depth studies to help establish whether there exists a direct causal and or inciting correlation between them. Understanding this association might lead to new research and treatment options for ACS patients.

## Introduction and background

In December 2019, the China Health Authority notified the World Health Organization (WHO) of cases of pneumonia with an unknown etiology in Wuhan, China. They identified the virus as a novel coronavirus, initially called 2019-nCOV. However, further research revealed that it was associated with the 2002 SARS-CoV-1 outbreak, and the virus was re-named SARS-CoV-2 by the International Committee for Taxonomy of Viruses [1,2]. On March 11, 2020, WHO declared SARS-CoV-2 infection a pandemic due to its rapid spread and increased mortality rate worldwide [3].

SARS-CoV-2 is a single-stranded RNA virus that causes the disease known as coronavirus disease 2019 (COVID-19) in humans. The condition is not limited to the respiratory system; studies have shown the manifestations of the infection seen in other body systems, including but not limited to the hematologic, gastrointestinal, and cardiovascular systems [4-6]. Research has linked the virus to major cardiovascular complications with or without respiratory symptoms. Furthermore, underlying comorbid cardiovascular disease (most commonly hypertension) has worsened the outcome in infected patients [7].

Acute coronary syndrome (ACS) encompasses unstable angina, non-ST-segment elevation (NSTEMI), and ST-segment elevation (STEMI). ACS is a sub-category of coronary heart disease which is the most prevalent cardiovascular disease. An estimated prevalence of 7.2% of the American adult populace aged 20 years or more has coronary heart disease [8]. The American Heart Association (AHA) estimates that 20.1 million American adults suffer from this disease, with one person suffering a myocardial infarction every 40 seconds [8].

The etiology of ACS among SARS-CoV-2 patients is not fully understood. While some experts have concluded on direct cardiac injury by the virus itself, others have described the breakdown of angiotensin-converting enzyme 2 (ACE2) as a possible mechanism of action and possibly, the rationale for the advent of the spike protein-based COVID-19 vaccine [9,10].

Among COVID-19 patients, particularly those requiring intensive care admission, laboratory findings of low lymphocyte count, elevated levels of cardiac troponin, interleukins, and procoagulant factors (prolonged prothrombin time and high D-dimer levels) have been found, which further buttresses a plausible link between COVID-19 and ACS [5,7,11].

This article will therefore discuss the pathophysiology of ACS as related to COVID-19, admissions, management guidelines of unstable angina (UA), NSTEMI, and STEMI, and the effect of the COVID-19 vaccine in preventing or reducing the impact of ACS.

## Review

Microbiology and clinical presentation of COVID-19

Coronavirus is an enveloped, single-stranded, positive-sense RNA virus that can cause respiratory diseases ranging from mild to lethal. They belong to the order Nidovirales, in the family Coronaviridae and have the largest genome of all RNA-based viruses [12-14]. The structure of the pathologic strains of COVID-19 includes the viral membrane, envelope, nucleocapsid, and spike proteins. Coronaviruses share these core conservative elements [15]. 

Interestingly, the three most notable coronaviruses affecting the human population are SARS-CoV-1, MERS-CoV, and the most recently discovered SARS-CoV-2 that caused the ongoing pandemic. In December 2021, there were five dominant variants of SARS-CoV-2 spreading among world populations: the Alpha variant, formerly called the United Kingdom (UK) variant, found in London and Kent; the Beta variant (formerly called the South Africa variant), the Gamma variant (formerly called the Brazil variant), the Delta variant (formerly called the India variant) and the Omicron variant [16-18].

Disease symptoms and modes of transmission are closely linked to inhalation of respiratory droplets when an infected person coughs, sneezes, talks, or exhales. Fundamentally, the main transmission route of coronavirus occurs through respiratory droplets exhaled by an infected individual; hence, inhaling these respiratory droplets predisposes a non-infected individual at risk of contracting the disease. Additionally, data has indicated that SARS-CoV-2 transmission can occur by contact with contaminated lifeless objects (fomite transmission) [19-21]. Disease severity ranges from mild to severe. Common symptoms include fever or chills, cough, shortness of breath or difficulty breathing, fatigue, sore throat, runny nose or congestion, diarrhea, body aches, and loss of taste and smell, with the latter being more specific to clinical diagnosis [22].

Pathophysiology of ACS

Acute coronary syndrome (ACS) is a term used when there is evidence or suspicion of myocardial injury. It is a broad spectrum of cardiac emergencies, ranging from UA, NSTEMI, and to the most life-threatening, STEMI. In the clinical setting, the diagnosis of acute myocardial injury is based on evidence of acute myocardial ischemia often presenting as left-sided chest pain and findings of abnormal cardiac enzymes including a rise in cardiac troponin levels above the 99th percentile of expected [23].

ACS is usually due to endothelial damage from atherosclerotic plaque build-up within the coronary vessels that help circulate oxygenated blood to the heart. Plaque formation leads to a complex sequence of an inflammatory state. Macrophages are released to engulf the lipid-laden plaque, trigger an inflammatory state, and cause endothelial dysfunction. Endothelial dysfunction leads to activation and aggregation of platelet microthrombi. This process triggers the release of tissue factors into the bloodstream, causing rupture and dislodgement of the plaque which obstructs cardiac blood flow [11,24]. 

**Figure 1:**
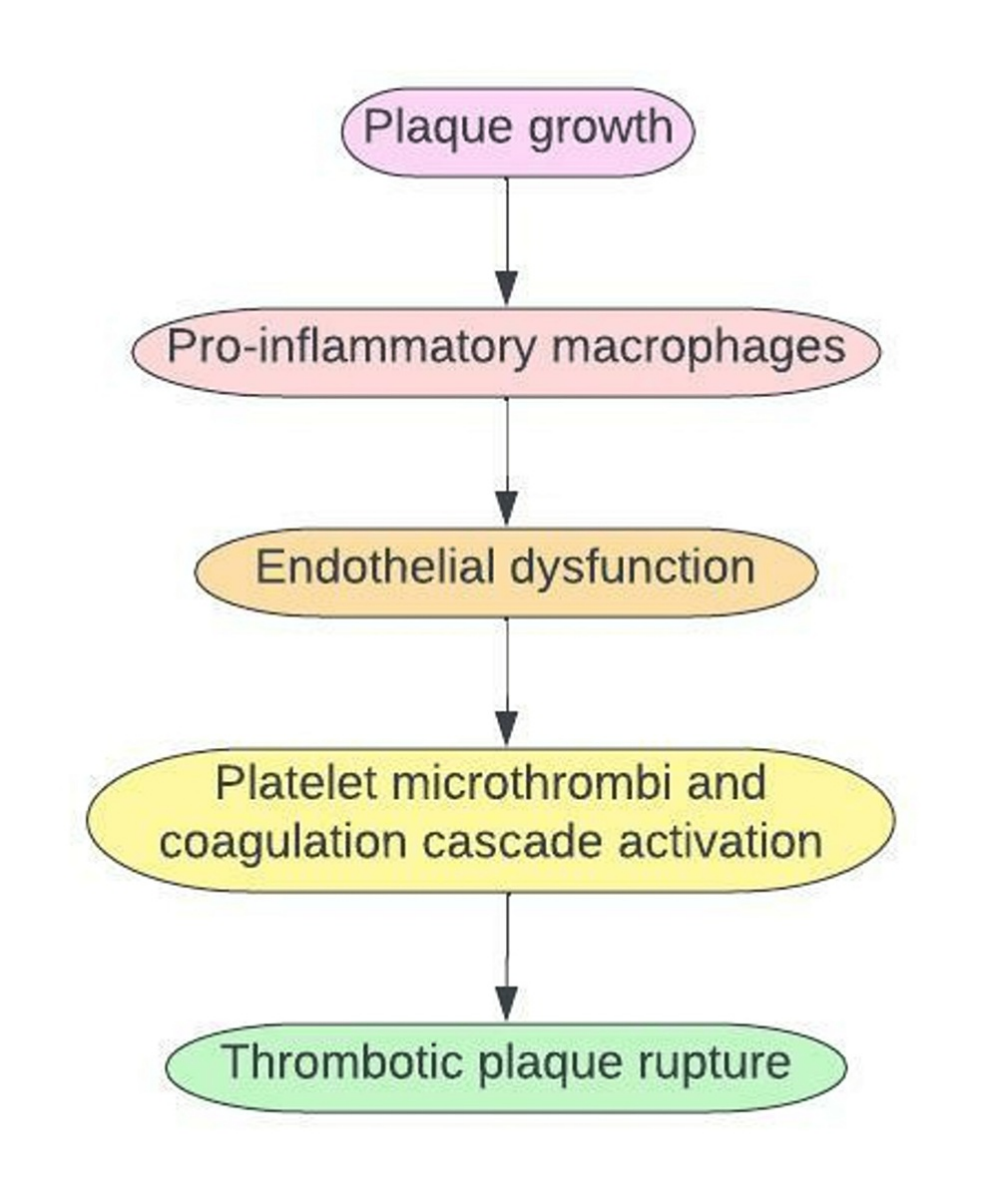
Pathogenesis of Acute Coronary Syndrome

The disease process of ACS was mainly associated with fatty (atherosclerotic plaque) endothelial damage previously, but studies have shown that ACS can occur even in the presence of normal coronary vessels. According to the current fourth universal definition of myocardial infarction, there are five subtype categories. The definition takes into account, the pathophysiologic events of acute coronary syndrome to include both plaque disruption from coronary atherothrombosis (type 1 MI) and myocardial injury in the absence of atherothrombogenic causes (type 2 MI) [23]. Type 2 MI occurs secondary to an ischemic process from increased or reduced myocardial demand for oxygen. Anemia, arrhythmia, and high inflammatory states such as sepsis are common major underlying causes of type 2 MI [25]. Hence, a strong correlation exists on the possibility of COVID-19-induced myocardial injury.

According to the American Heart Association, coronary flow obstruction typically causes myocardial demand and oxygen supply mismatch, but other non-flow coronary causes have also been identified [26]. The allusion to the association of the pathogenesis of ACS with a multifactorial cause beyond a flow obstruction becomes even more pertinent in evaluating a possible causal relationship between the emergent SARS-CoV-2 and ACS. The entirety of the mechanism leading to ACS in SARS-CoV-2 infection is not yet fully understood, but the virus has been studied and found to have a very high binding affinity and cleavage pattern of ACE2 in facilitating entry and replication within the human host cell. Hence, the development of a spike protein-based vaccine may depend on this pathway as a treatment prevention strategy for viral replication [9,10].

Angiotensin II, a cleavage product of ACE2 and a potent vasoconstrictor, leads to the formation of reactive oxygen species (ROS) that can potentiate coronary blood vessel wall inflammation and subsequent development of ACS. Thus, ACE2 is a prognostic marker of disease in patients with COVID-19, especially among those with underlying co-morbid conditions such as hypertension and diabetes [27].

Notably, the existing viral (e.g., influenza) pathogenic pattern of effect on coronary vessel inflammation with the release of cytokines leading to endothelial dysfunction is one of the mechanisms of action postulated to be a cause of ACS among COVID-19 patients. As with other cases of viral infection, the cytokine release syndrome, which occurs as part of the innate immune response, has also been observed in cases of severe COVID-19 disease and has been shown to cause the systemic release of inflammatory cytokines such as interleukin 1B, 2, 6, 8, 10 and tumor necrosis factor-alpha [11].

Increased C-reactive protein (CRP) levels, cytopenia, prolonged prothrombin time, elevated D-dimer values, and high troponin levels can be evaluated for and seen in severe cases of COVID-19 patients in particular. End-organ damage, including endothelial cell dysfunction and STEMI, can result from the pro-inflammatory and procoagulant state [7,24,28]. According to a retrospective study conducted in Guizhou, China, elevated levels of CRP, an acute-phase reactant and a marker of inflammation, is a direct marker of increasing severity in COVID-19 patients. The study established that reactant protein is a cause of endothelial dysfunction and also accelerates the process of atherosclerosis with an elevated increase in the production of adhesion molecules within the endothelial cells. CRP is also known to inhibit the production of both nitric oxide and prostacyclin by endothelial cells, further supporting its role in the thrombotic plaque formation of ACS [29,30].

The final common pathway with high surge release of catecholamines, procoagulant factors, hypoxia, and elevated levels of proinflammatory markers in COVID-19 patients can lead to thrombotic plaque rupture leading to an ischemic event [24].

COVID-19 induced ACS 

Despite compounding evidence establishing a possible causal relationship between COVID-19 infection and ACS, there are currently no sufficient data to categorically state that the advent of ACS observed among this subset of patients is due to an underlying medical condition or as a result of a direct cardiac effect of the virus itself. The overlapping laboratory evidence such as troponin I and clinical features of chest pain and dyspnea seen in both cases of myocarditis and myocardial infarction can also pose as a limiting factor in diagnosis [31,32].

According to a study carried out among 41 confirmed adult cases in Wuhan, China, 12% had COVID-19 complicated acute cardiac injury in which troponin I, a sensitive marker of ACS, was substantially high [5]. Prothrombotic factors such as prothrombin time and D-dimer value were significantly high upon hospital admission, particularly those requiring intensive care unit (ICU) management. This set of patients requiring critical care also demonstrated significantly lower left ventricular ejection fraction as demonstrated by their transthoracic echocardiograph [5,33]. 

Another retrospective study conducted in India among 511 patients who tested positive for SARS-CoV-2 showed that the most common cardiac complication, especially with COVID-19 hospitalized patients, including those with previous history (150 patients) of pre-existing ischemic heart disease was the development of ACS. The observed cases of ACS in this study were defined according to the fourth universal definition of myocardial infarction. Hence, elevated levels of troponin above the 99th percentile of the upper limit were used in case selections. Results of the study showed STEMI in 161 patients (31.5%) and NSTEMI in 99 patients (19.4%) [34].

Since the COVID-19 pandemic was declared, a substantial reduction in the admission rates of ACS was surprisingly observed despite a strong correlation with the infection. There was an almost 50% reduction in the hospitalization rates in patients with NSTEMI during the lockdown period. A study done in Germany showed a 23% decrease in admission rates for UA. In contrast, the rate of admitted STEMI patients was stable [35-37]. Similarly, another retrospective cross-sectional analysis carried out in Pakistan revealed decreasing UA and stable STEMI admissions. However, a comparative analysis of the Pakistan study demonstrated an upward trend in the admission rate of NSTEMI patients [38].

In India, an observational study found a decline in admission rates amassing 43% between the period of March 25 and May 31 of 2020 when compared to the same time frame two years prior. This study demonstrated an overall decrease in hospital admission of all three types of ACS [39].

Interestingly, one would have expected an increase in the use of percutaneous coronary intervention (PCI) for an apparent number of reasons such as heightened psychosocial stressors and viral-induced STEMI during the COVID-19 period. A confounding paradox was the reduction in the use of invasive procedures relating to STEMI during the early phase of the COVID-19 era. There was a 38% and 40% reduction in cardiac catheterization in the United States and Spain respectively [40].

Stressful physical activity, exposure to polluted air, and other respiratory diseases such as the flu, which are myocardial infarction (MI) triggers, were reduced during the lockdown. The aforementioned factors were attributed as probable reasons for the patients’ decline in ACS hospitalization [41,42]. A study in Austria proposed that the reduction in observed cases of COVID-19-induced ACS admissions was due to the overburdened healthcare system, a factor significantly more apparent during the COVID-19 pandemic. As a result, ischemic episodes were significantly underdiagnosed [41].

Management of ACS in COVID-19 patients

ACS is a medical emergency that requires a timely diagnosis and intervention; hence specific guidelines vary according to the patient’s severity upon admission. When selecting the appropriate management strategy, it is essential to consider the clinical status of a patient presenting with ACS and the risk of co-existing COVID-19 infection. The recommendation is that all patients be managed as COVID-19 positive until their test results are known [43].

As soon as a patient presents to the emergency department with clinical features of ACS and a baseline electrocardiogram, commencing medical therapy should be the next step before deciding on invasive therapy. The initial medical treatment closely mirrors that used in patients without COVID-19. Medical treatment may include dual antiplatelet medications: aspirin and a P2Y12 inhibitor such as clopidogrel which is considered only when invasive management will be delayed and the patient is not at risk of bleeding; beta-blockers if there are no contraindications; angiotensin-converting enzyme inhibitors/angiotensin receptor blockers; nitrates (except with right ventricular infarction in which decreasing preload may be harmful) and statins. If hypoxia is present, the patient should receive oxygen [44,45]. 

Unstable Angina (UA)

Within the first hour of the presentation, it is crucial to identify a patient with UA as immediate management is targeted at symptomatic relief of chest pain using nitrates. The treatment approach also includes the use of the anti-platelet medication; aspirin which acts by inhibiting the cyclooxygenase pathway. The use of anti-platelet agent such as clopidogrel which is a P2Y12 inhibitor is also permitted on a case-by-case basis in combination with aspirin particularly if there are no risks of bleeding and invasive therapy is non-emergent [46].

According to the European Association of Percutaneous Cardiovascular Interventions (EAPCI), an invasive procedure for unstable angina was categorized as urgent and may be performed within days of presentation. The timing of intervention may be affected by the overwhelming demand and resources in the setting of the COVID-19 pandemic [43].

One of the many case examples is that of a 70-year-old man with a newly diagnosed COVID-19 infection and a background history of unstable angina. Initial management included transfer to a negative pressure airflow room, use of aspirin, and atorvastatin. The worsening decline in respiratory function prompted the use of an urgent percutaneous invasive therapy to prevent worsening morbidity [47].

Non-ST-Elevation Myocardial Infarction (NSTEMI)

Management of NSTEMI may require the use of either medical or early (<24hr) invasive therapy (percutaneous coronary intervention) [48]. A study by Liu et al. in Beijing however recommended that treatment for NSTEMI during the pandemic should be determined based on risk stratification and that patients with extremely high-risk clinical presentations such as hemodynamic instability and refractory chest pain should undergo invasive therapy within two hours of their initial presentation [49]. Unfortunately, actual clinical case scenarios using the recommended two-hour timeline proved to be an otherwise arduous task due to the overwhelming influence of COVID-19 on the health system. Invasive therapy within the proposed timeline is vital among the very high-risk NSTEMI patients because those managed with conservative treatment during the COVID-19 pandemic had more significant adverse effects in comparison with pre-pandemic patients having a similar NSTEMI risk stratification [49].

It is recommended that NSTEMI patients should be treated in the same manner irrespective of whether suspected or confirmed cases, with a diagnosis of COVID-19 obtained as soon as possible [50]. Low or intermediate-risk NSTEMI patients with suspected or proven COVID-19 infection should receive optimized medical treatment in intensive care unit (ICU) quarantine wards and, if necessary, PCI once the quarantine period expires. After optimized medical treatment, high-risk or extremely high-risk patients should receive early (<24hr) or immediate (<2hr) PCI, respectively [50].

ST-Elevation Myocardial Infarction (STEMI)

Currently, there is no single international management guideline consensus regarding the choice of initial reperfusion therapy in STEMI occurring among COVID-19 patients. Following specific health institution guidelines, this decision lies with the interventional cardiologist [51].

According to the American Heart Association, in the case of suspected infection, testing for COVID-19 should not delay primary PCI in patients with STEMI, and PCI should continue to be the primary re-perfusion method for STEMI patients because it has a superior outcome to other therapeutic options [52]. The EAPCI recommended that if primary PCI is not possible within 120 minutes (door to balloon time), fibrinolytic treatment should be the first-line alternative if not contraindicated [43]. However, in some centers, thrombolysis was used as the first-line treatment. A study in the United Kingdom showed the use of thrombolysis in managing STEMI, with only one-third of their patients undergoing PCI. This latter category experienced significant delays in interventional therapy [53].

A study in Liaoning, China, also advised thrombolysis as a first-line treatment for STEMI patients with suspected or confirmed COVID-19 infection and symptoms that appear within 12 hours. PCI, performed in a conventional catheter lab, was recommended in these places only if thrombolysis is not an option or if it fails. If the onset of symptoms is beyond 12hrs, optimized medical treatment in ICU quarantine wards is ideal. COVID-19 should be ruled out immediately with a lung computed tomography (CT) scan and SARS-CoV-2 nucleic acid testing, followed by PCI in a routine catheter lab for patients that meet the criteria [50].

Patients in Lithuania, Italy, and Iraq who had STEMI with concomitant COVID-19 and were hemodynamically stable were also treated with fibrinolytic therapy first. When the treatment was successful, patients were initially discharged home with a subsequent return for invasive revascularization when they tested negative for COVID-19 at least 14 days after their diagnosis [51].

Cardiac effects of COVID-19 vaccine

To prevent continuous transmission of SARS-CoV-2 and reduce the disease burden worldwide, scientists have investigated the development of different vaccine types at various levels. Following the onset of the COVID-19 pandemic in late 2019, various therapeutic regimens such as nucleoside analogs, IL-6 inhibitors, immunotherapy, and herbal medicine have been developed. Currently, utilized formulations are mainly viral and protein-based [54].

Out of the vaccines approved by the Food and Drug Administration (FDA), Pfizer-BioNTech, Moderna, and Janssen have demonstrated significant efficacy and safety in clinical trials without any observed major adverse effects. However, new reports raise the potential association in developing adverse cardiac effects following the administration of the aforementioned COVID-19 vaccines [55]. Regulatory surveillance and self-reporting systems such as the Vaccine Adverse Events Reporting System (VAERS) in the United States, the Yellow Card System in the United Kingdom, and the EudraVigilance system in Europe have linked several COVID-19 vaccines currently in use to possible cardiovascular side effects [56].

A possible underlying obstacle in early recognition of any potential acute cardiovascular side effects may be difficult to ascertain. Post-vaccine injection symptoms such as site soreness and other non-major local or systemic reactions, including perceived pain at the injection site, erythema, swelling, fever, headache, and myalgia, may be confused and overlooked by the patient as non-cardiac ischemic symptoms, hence, resulting in delayed presentation or findings of resolved old MI. Various cardiac complications, particularly for messenger RNA (mRNA) based COVID-19 vaccines, may range from acute myocardial infarction, pulmonary embolism, and stroke to venous thromboembolism [54,57].

COVID-19 mRNA vaccines, such as mRNA1273 (Moderna vaccine) and BNT1626b2 (Pfizer-BioNTech vaccine), induce innate immunity, T cells (cytotoxic and helper T cells) as well as B cell responses specifically. Also, the mRNA vaccine is said to increase interleukin 16, which is a proinflammatory cytokine, soluble Fas (an inducer of apoptosis), and Hepatocyte Growth Factor (HGF), which serves as a marker for chemotaxis of T-cells into the cardiac tissue and are responsible for initiating the inflammatory process within the endothelium and activation of a microthrombi, thereby resulting in coronary wall ischemia [54,58].

Another newly established phenomenon is that of vaccine-induced immune thrombocytopenia and thrombosis (VITT) which manifests as atypical thrombosis as well as thrombocytopenia following COVID-19 vaccine immunization. Although the reported incidence is very low and has minimal hindrance to the overall benefit of immunization, VITT can lead to fatal complications [59,60]. VITT is usually observed within four to 28 days post-vaccination and one case report of VITT-induced myocardial infarction has been observed in a 75-year-old female with end-stage renal disease eight days after receiving the Oxford-AstraZeneca COVID-19 vaccine [61].

A study that found a temporal relationship between COVID-19 vaccinations and adverse cardiac events revealed that patients who developed cardiovascular manifestations after receiving the vaccine mainly were men. Of this category, those who developed myocarditis were younger and more likely to present three days post-vaccination. In contrast, those who developed acute myocardial infarction were older and more likely to present 24 hours after the first dose of the vaccine [55].

According to Boivin et al., a 96-year-old female patient experienced acute MI after receiving the first dose of the Moderna vaccine. Still, the analysis failed to establish a direct causal link between the vaccine and the precipitation of the cardiac event. Another case series from Singapore also reported cardiovascular-related symptoms and biochemical and electrocardiographic evidence of myocardial injury among three previously healthy patients shortly after receiving the Pfizer-BioNTech COVID-19 vaccine. Despite the findings of these isolated events, there are currently no ground-breaking research studies to prove beyond reasonable doubt that occurrences of ACS are directly related to the vaccine itself [57,62].

While the COVID-19 vaccine is primarily safe and effective in reducing the transmission of SARS-CoV-2, its cardioprotective function is yet to be ascertained, unlike the established protective effect of the viral influenza vaccine on the heart. Undisputedly, there is a lot to be unraveled about the novel virus and points to specific areas of research focus in the nearest future [63-68].

## Conclusions

Given the relevance of ACS in clinical settings, a possible association with COVID-19 significantly exacerbates the unfavorable clinical outcomes of those affected by both conditions simultaneously. Notably, this association increased mortality, morbidity, and hospitalization rates, and reduced quality of life.

Despite the currently observed outcomes, COVID-19 is a relatively new subject with extensive ongoing research and thus, limits currently available knowledge of this infectious disease. There is still a need for more in-depth studies to help establish a direct causal and or inciting relationship between COVID-19 and ACS, as well as to construct a standard algorithm in diagnosis and management that can serve as a high-yield resource tool for clinicians and health-related allies.
